# Five Cases of Injury to Main Vessels Occurring in Civil Practice

**Published:** 1930

**Authors:** Robert V. Cooke

**Affiliations:** Assistant in the Surgical Unit, The Welsh National School of Medicine, Cardiff


### FIVE CASES OF INJURY TO MAIN VESSELS OCCURRING IN CIVIL PRACTICE.

BY

Robert V. Cooke, M.B., Ch.M. Bristol., F.R.C.S. Eng.,

*Assistant in the Surgical Unit,*
*The Welsh National School of Medicine, Cardiff.*

---

The following five cases of injury to main vessels would appear to be interesting, and they serve to emphasize the value of some simple and well-known surgical procedures :—

**Case 1.**—W. L., male, aged 38. The right forearm was crushed while he was at work in a mine. He continued work, but by night the arm had become very painful. He noticed that it was " swollen and hard." Pain continued all night, and next day he could not move his wrist and fingers. When he came to the Cardiff Royal Infirmary his right forearm was very swollen and very tense and particularly hard on the flexor aspect. The skin was unbroken, and there was no evidence of bruising. The right hand was slightly swollen. It was pale, and exhibited purplish mottling in places. No pulse could be felt at the wrist. Only the very slightest movement of the wrist and fingers was possible. The fingers were anæsthetic.

On the way to the theatre an X-ray was taken. There was no bony injury. Lengthy incisions were made on the flexor aspect of the forearm, and as soon as the deep fascia was incised masses of clot escaped—obviously under extreme tension. When some of the clot had escaped free hemorrhage followed. It was found that the ulnar artery had been torn at the origin of a muscular branch. Although the muscles tended to bulge through the incisions, no attempt to suture the wounds was made, but they were left widely open.

Within an hour a faint pulse could be felt at the wrist. By next morning the radial pulse was quite good and more movement was possible in the fingers. Blood-clot came away for over a week, and then the wounds healed. After a fortnight there was a slight persistent flexion at the wrist and fingers. Now, after three months' massage and movements, together with every co-operation from an intelligent patient—anxious to get well—the wrist and finger movements are almost as good as ever. He has resumed his usual occupation.

Without bony injury or open wound it is unusual for a large vessel to be ruptured by a moderate crushing violence. Only a speedy intervention prevented much damage to the hand from a combination of extreme tension and the resulting ischæmia. The importance of the attitude of the patient and his co-operation towards a satisfactory result was very evidently demonstrated.

**Case 2.**—W. M., male, aged 19. He was struck at the back of the left knee by a tram of coal. Next day, because of pain, swelling and bruising, he was brought to the Casualty Department, Cardiff Royal Infirmary. An X-ray showed no bony injury, and the boy was referred back to his panel doctor.

A week later the case was admitted as an emergency. The boy was obviously very ill, with a high temperature and rapid pulse. There was still some bruising and a slight swelling at the back of the knee. The knee was moderately flexed and could not be fully extended. There was gangrene of the right foot spreading up the leg. The history was that three days after the injury there was pain in the foot and cramp-like pains in the leg. Then the foot had begun to become discoloured, and for two days it had lost all sensation and become black. It was apparent that what was originally a dry gangrene was now a rapidly-spreading moist gangrene. It was felt that an attempt should be made to save the knee joint, although the chances of healing of an amputation below the knee were small. There was also the fixed flexion at the knee which was likely to give trouble with the fitting of an artificial limb to the stump.

A mid-tibial amputation was done. Although the usual tourniquet was discarded, there was practically no bleeding when the tibial vessels were cut because they were thrombosed. The flap partly sloughed, and some infection of the wound occurred. Later a trimming operation was done, and now the stump is soundly healed. During the procedures every effort was made to preserve mobility and function of the stump. Flexion—present on admission—has become less and less, and now, as the result of the patient's perseverance, the flexion has been practically overcome.

The case was regarded as one of thrombosis of the popliteal artery following a trauma. There was never enough swelling for an actual rupture of the main vessel to have taken place. Probably what swelling there was in the neighbourhood of the knee contributed towards the failure of the collateral circulation, which is, in any case, somewhat precarious. The interference with the circulation at the back of the knee would also appear to have given rise to a degree of ischæmic contracture. Needless to say, the end result was quite unlooked for on the part of the resident, who discharged the case as one of mere bruising.

**Case 3.**—E. L., male, aged 22. He was received into the Royal Free Hospital observation ward after a motor-cycle accident. There were superficial leg wounds and evidences of a closed fracture. The wounds were dressed and the leg put in box splints. Extreme pain was experienced all night. Next day an X-ray examination showed a comminuted fracture of right tibia and fibula. Pain in the leg was still intense, and the removal of splints showed a very tense, swollen limb. There were blebs over the whole limb, which was purplish-black in colour and obviously on the point of gangrene. The foot was cold, and no pulse was palpable in it.

As soon as possible long incisions were made to relieve the tension, which was jeopardizing the life of the limb. Extensive intramuscular hemorrhage had occurred, and when large masses of clot had escaped arterial bleeding became profuse. When a tourniquet had been applied a torn anterior tibial artery was discovered and tied. At the site of fracture a fragment of tibia was seen to be almost loose from any connection with muscle or periosteum. This was removed. Extension being necessary, skeletal traction was employed (on a pin through the os calcis). The state of the skin and the extent of the operation wound precluded any other form of satisfactory extension being used.

A certain amount of soft tissue sloughed, and it was not surprising that infection occurred. This fact, together with the gap produced by the removal of bone, resulted in a very delayed union. Two years later the man had a good, sound limb.

The case illustrates one of the less common complications of a simple fracture. Early operation was imperative in order to save the limb from the effects of extreme tension. Even so, operation was too long delayed in this case. The case also serves to emphasize once again the value of skeletal traction, and that in the débridement of open fractures the utmost conservatism should be observed in the matter of removal of bone fragments.

**Case 4.**—B. M., male, aged 47.—While he was at work in a mine an explosion caused a large piece of stone to shatter his left femur and produced a large ragged wound on the inner aspect of his thigh from which there was at once dangerous hemorrhage. When seen a few hours later the bleeding had ceased, but the general condition was desperate. The dorsalis pedis artery on the right side could not be felt. There was no anæsthesia of the leg or foot. He rallied a little later the same evening, and under light anæsthesia the leg, which showed gross deformity, was quickly pulled out straight and temporarily extended by means of a skin traction apparatus and a Thomas's splint. Straightway the dorsalis pedis artery could be felt to pulsate. The large wound led down to the popliteal surface of the femur, which was felt to be much comminuted. The popliteal artery was not felt. No attempt was made to excise the wound. It was lightly packed with gauze soaked in paraffin and flavine. Antitetanic serum was given.

On the second day after the accident the wound showed signs of infection, and at the same time the circulation in the foot appeared precarious. On the third day there was no pulse palpable in the foot, which showed discoloured areas and had become almost anæsthetic. On the fourth day gangrene had set in the foot and the thigh wound was suppurating.

Amputation was done through the thigh well above the wound. Even so there was pus in the subcutaneous tissues. The flaps were left almost unsutured. Suppuration delayed healing, but this has now occurred. Dissection of the amputated limb showed that at the site of the initial injury the popliteal vessels were bruised and thrombosed.

A grossly contaminated compound comminuted fracture is in itself a very serious injury, and on that score alone immediate amputation would have been indicated were it not for the desperate condition of the patient. Later thrombosis of the main artery and gangrene necessitated amputation. Again close co-operation by a patient desirous of a good result has resulted in the maintenance of full movements and power in a useful stump.

**Case 5.**—W. L., male, aged 38. Whilst drunk he staggered against a moving tram, which struck him in the right axilla. He was admitted to Royal Free Hospital with severe bruising about the right shoulder. The skin over the inner aspect of the upper arm was intact, so also was the humerus, but the intervening soft parts were apparently pulped. The hand and forearm were cold, insensitive and pulseless. Immediate exploration of the third part of the axillary artery showed that whilst the main vessels and nerves were not actually severed, they were lying in a bruised condition in a mass of blood clot. All the muscles in the vicinity were pulped. The artery was thrombosed for a length of many inches. It was hoped that collateral circulation might become established, although such extensive damage well-nigh precluded this possibility.

Next day the hand was stiff, cold and insensitive, gas-gangrene supervened, and in a few hours the muscles of the pectoral girdle were grossly involved. Amputation at the shoulder was performed, large masses of infected muscles were removed and the flaps left widely open. The man's condition was desperate, but with the aid of blood transfusions and large quantities of anti-gas-gangrene serum, he gradually recovered. The truly immense wound was dressed without pain with large gauze packs soaked in olive oil containing $2\frac{1}{2}$ per cent. carbolic acid. Later the flaps were sutured, and complete healing was furthered by plastic procedures.

The case provides yet another example of the danger of temporizing with a severely-crushed limb. It must always be a nice point to decide between immediate amputation and a trial of conservative measures. Few would overlook the smallest chance of saving a right upper limb.

I am indebted to Mr. C. A. Joll, to Mr. P. Jenner Verrall and to Professor A. W. Sheen for allowing me to report these cases.

